# Two New Lytic Bacteriophages of the *Myoviridae* Family Against Carbapenem-Resistant *Acinetobacter baumannii*

**DOI:** 10.3389/fmicb.2018.00850

**Published:** 2018-04-30

**Authors:** Weilong Zhou, Yu Feng, Zhiyong Zong

**Affiliations:** ^1^Center of Infectious Diseases, West China Hospital, Sichuan University, Chengdu, China; ^2^Division of Infectious Diseases, State Key Laboratory of Biotherapy, Chengdu, China; ^3^Department of Infection Control, West China Hospital, Sichuan University, Chengdu, China; ^4^Center for Pathogen Research, West China Hospital, Sichuan University, Chengdu, China

**Keywords:** bacteriophage, phage therapy, carbapenem resistance, *Acinetobacter baumannii*, *Galleria mellonella*

## Abstract

Two lytic bacteriophages, WCHABP1 and WCHABP12, were recovered from hospital sewage and were able to infect 9 and 12 out of 18 carbapenem-resistant *Acinetobacter baumannii* clinical strains, which belonged to different clones. Electron microscopy scan showed that both bacteriophages had the similar morphology as those of the *Myoviridae* family. Whole genomic sequencing revealed 45.4- or 45.8-kb genome with a 37.6% GC content for WCHABP1 and WCHABP12, both of which showed significant DNA sequence similarity with bacteriophages of the *Ap22virus* genus within the *Myoviridae* family. Taxonomic analysis was therefore performed following the proposal approved by the International Committee on Taxonomy of Viruses, which confirmed that WCHABP1 and WCHABP12 represented two new species of the *Ap22virus* genus. No tRNAs but 88 and 89 open reading frames (ORFs) were predicted for the two bacteriophages, among which 22 and 21 had known function and encoded proteins for morphogenesis, packaging, lysis, and nucleiotide metabolism. The C-terminal amino acids of the large unit of fiber tail proteins varied between the bacteriophages, which may explain their different host ranges. For most lytic bacteriophages, a set of holin and endolysin are required for lysis. However, no known holin-encoding genes were identified in WCHABP1 and WCHABP12, suggesting that they may use alternative, yet-to-be-identified, novel holins for host cell membrane lysis. To test the efficacy of the bacteriophages in protecting against *A. baumannii* infection, a *Galleria mellonella* larva model was used. Only <20% *G. mellonella* larvae survived at 96 h after being infected by carbapenem-resistant *A. baumannii* strains, from which the two bacteriophages were recovered. With the administration of WCHABP1 and WCHABP12, the survival of larvae increased to 75%, while the treatment of polymyxin B only slightly increased the survival rate to 25%. The isolation of two new lytic bacteriophages in this study could expand our sight on *Acinetobacter* bacteriophages and may offer new potential therapeutic alternatives against *A. baumannii*.

## Introduction

On 27 February 2017, World Health Organization (WHO) released a list of 12 types of antimicrobial-resistant “priority pathogens” that pose the greatest threat to human health (World Health Organization, 2017). In the list, *Acinetobacter* along with *Pseudomonas* and various species of the Enterobacteriaceae (e.g., *Klebsiella, Escherichia coli, Serratia*, and *Proteus*) are labeled as “Critical,” for which new antimicrobial agents are urgently needed. *Acinetobacter baumannii* is a non-fermentative, non-motile, catalase-positive, gram-negative bacterium. It is widely dispersed in soil and water as well as the healthcare environment with the ability to cause various infections such as bacteremia and pneumonia (Dijkshoorn et al., 2007; Peleg et al., 2008). In addition to its capability of survival on dry and moist surfaces (Berlau et al., 1999), a variety of intrinsic and acquired mechanisms for antimicrobial resistance and virulence (Di et al., 2011; Perez et al., 2011) have made *A. baumannii* a successful pathogen worldwide (Perez et al., 2007).

One century ago, since the first day of their discovery, the compelling feature of bacteriophages as a natural enemy of bacteria has drawn attention of early researchers to exploit them as a mean to treat bacterial infections (d'herelle, 1917; Bruynoghe and Maisin, 1921; Helvoort, 2001). However, the successive discovery of penicillin and other antibiotics soon put bacteriophages on the shelf, with exception in the Soviet Union and Poland (Kutter et al., 2015). The emergence of multi-drug resistant (MDR) bacteria calls for alternatives of antibiotics and bacteriophages therefore gain interests for extensive studies again (Smith and Huggins, 1983; Soothill, 1992).

As a potential alternative to antibiotics and a possible solution for impending infections caused by MDR bacteria, there are still constraints for bacteriophages to be widely applied in real clinical practice despite the presence of reported successful cases (Abubakar et al., 2016). The main challenges for such a bacteriophage therapy include that the relatively narrow lytic range against bacterial strains and the proneness of bacteria to develop resistance. To tackle the challenges, multiple lytic bacteriophages have been used in combination as cocktails (Merabishvili et al., 2009). The success of cocktail bacteriophage therapy against predominantly MDR bacteria such as *A. baumannii* relies on isolation of novel lytic bacteriophages. Here we report two newly isolated lytic bacteriophages named WCHABP1 and WCHABP12 of the *Ap22virus* genus within the *Myoviridae* family against carbapenem-resistant *A. baumannii* clinical isolates.

## Methods

### Bacterial strains

This study included a total of 18 nonduplicate carbapenem-resistant (MICs of imipenem ≥ 8 mg/L) but polymyxin B-susceptible (MICs <2 mg/L) *A. baumannii* clinical strains recovered from blood, sputum, urine, ascites, bronchoalveolar lavage fluid (BALF), and drainages of different patients at West China Hospital, Sichuan University, China, from April to June 2016 (Table 1) as hosts for bacteriophage isolation. The species identification of them was established by partially sequencing the *recA* gene as described previously (Krawczyk et al., 2002). These isolates belonged to 18 different clones as determined using a pulsed field gel electrophoresis (PFGE) analysis (data not shown). Clones were defined for isolates with an 80% or above pattern similarity. Antimicrobial susceptibility testing was performed using the Vitek II automated microbiological system (bioMérieux, Marcy-l'Étoile, France) or broth microdilution method of the Clinical and Laboratory Standards Institute (CLSI) (CLSI, 2017) (for colistin, imipenem, polymycin B, and tigecycline). Breakpoints defined by U. S. Food and Drug Administration were used for tigecycline; otherwise, those defined by CLSI were applied. The results are shown in Table S1.

**Table 1:** Host ranges of WCHABP1 and WCHABP12.

**Strain**	**Source**	**MICs (mg/L)**			**EOP**	
		**Imipenem**	**Colistin**	**Tigecycline**	**WCHABP1**	**WCHABP12**
Ab1138	Blood	64	1	1	0.2	
Ab1186	Ascites	64	1	0.5	1^+^	0.8
Ab1262	Sputum	64	1	0.5		1^+^
Ab1334	BALF	16	0.5	0.5		
Ab1337	Sputum	64	1	0.5	0.6	
Ab1369	Sputum	64	1	0.5		
Ab1391	Sputum	64	1	0.5		0.8
Ab1397	Sputum	64	1	0.5		0.8
Ab1412	Ascites	64	1	0.5		0.8
Ab1415	Sputum	64	1	0.5	0.1	0.3
Ab1454	Urine	64	1	0.5		
Ab1478	Blood	64	0.5	0.5		0.1
Ab1497	Sputum	64	1	0.5	0.7	0.6
Ab1531	Sputum	32	1	0.5	0.8	0.6
Ab1585	Sputum	16	1	1	0.5	0.8
Ab1588	Ascites	16	0.5	1		
Ab1623	Blood	64	0.5	1	0.8	0.2
Ab1673	Blood	64	0.5	1	0.4	0.3

+ *The original host strain, from which the bacteriophage was recovered*.

### Bacteriophage isolation, titering, and concentration

Bacteriophages were isolated from sewage samples collected at the influx of the wastewater treatment plant at West China Hospital in September 2016. The isolation and propagation of bacteriophages were performed as described previously (Merabishvili et al., 2009) and titering was carried out using the agar overlay method (Kropinski et al., 2009). PEG 8000/NaCl precipitation was used to concentrate bacteriophages for further use as described previously (Peng et al., 2014).

### Determination of the host range

The bacteriophage stock was diluted 1:10^6^ with Luria-Bertani (LB) broth (Hopebio, Qingdao, China) and was adjusted to a final titer of 1 × 10^4^ pfu/ml. Aliquots of 10 μl diluted bacteriophages were mixed with tested bacterial host strains or the original strains from which the bacteriophages were isolated. The mixtures were subjected to agar overlay method. The efficiency of plating (EOP) was calculated by dividing the bacteriophage titer of tested strain by that of original strain. A particular bacteriophage-bacterial strain combination was considered as high, medium and low production efficiency if the average EOP value was ≥ 0.5, ≥ 0.1 to < 0.5, and ≥ 0.001 to < 0.1, respectively; while a < 0.001 average EOP value suggests inefficient production (Khan and Nilsson, 2015).

### Electron microscopy

The concentrated bacteriophage stocks were negatively stained with phosphotungstic acid (PTA) and were then imaged using a H-600 II transmission electron microscope at 75 kV (Hitachi, Tokyo, Japan) to acquire morphological information of single bacteriophage particles.

### Multiplicity of infection (MOI) assay

Bacteriophage stocks were diluted by LB broth (Hopebio, Qingdao, China) into 10-fold series. Aliquots of each dilution were mixed with their corresponding host cultures at 10^8^ cfu/ml to result in different ratio (from 10^−4^ to 10 pfu/cfu) and were incubated at 37°C for 4 h. Bacteriophage progenies of each mixture ratio were titered to determine the highest production as the optimal MOI (Peng et al., 2014).

### Adsorption rate assay

Aliquots of bacteriophages were incubated with host strain cultures at the optimal MOI under 37°C. Samples (100 μl) were collected at 0, 3, 6, 9, 12, and 15 min, respectively, and were centrifuged to remove the bacterial cells. The supernatants were titered for determination of unabsorbed bacteriophages at each time interval (Peng et al., 2014).

### One-step growth curve

Bacteriophages (10 μl at a titer of 1 × 10^9^ pfu/ml) were mixed with 5 ml of their host strain cultures at about 2 × 10^7^ cfu/ml to reach a MOI of 0.1. The mixture was incubated at 37°C for 10 min to allow the complete adsorption and was then centrifuged at 15,000 g for 1 min to remove the unabsorbed bacteriophage particles by discarding the supernatants. The pellets were resuspended in LB broth and were incubated at 37°C. Aliquots (100 μl) were taken every 10 min from the beginning to 90 min and were titered for free bacteriophages after being centrifuged at 15,000 g for 1 min to remove bacterial cells. An additional 100 μl aliquot was taken at the beginning (0 min). A half of the aliquot (50 μl) was centrifuged and the remaining half was not centrifuged, both of which were titered for bacteriophages. Infected cells were calculated by subtracting the number of free bacteriophages of the centrifuged part from the number of bacteriophages of the un-centrifuged part, then the burst size could be calculated as dividing the maximal progeny counts by the number of infected cells (Peng et al., 2014).

### Bacteriophage genome sequencing and bioinformatics analysis

Bacteriophage DNA was prepared using the standard phenol-chloroform extraction (Mandell and Hershey, 1960) after concentration. Whole genome sequencing was performed using the HiSeq 2500 Sequencer (Illumina, San Diego, CA, USA) with 150-bp paired-end (the final coverage was ~200x). *De novo* assembly was performed using SPAdes v3.10.1 (Bankevich et al., 2012) with auto-cutoff and careful mode. Annotation of the genomic sequence was carried out using Prokka v1.11 (Seemann, 2014) followed by manually confirmation via running BLASTp and PSI-BLAST (http://blast.ncbi.nlm.nih.gov/) (Altschul et al., 1990, 1997) against the non-redundant protein database with a significant E-value of <10^−3^. For annotated proteins, the conserved protein domains/motif and additional function inference were detected using InterProScan (http://www.ebi.ac.uk/interpro/search/sequence-search) (Quevillon et al., 2005), HMMER (https://www.ebi.ac.uk/Tools/hmmer), and Conserved Domain Database (https://www.ncbi.nlm.nih.gov/cdd) (Marchlerbauer et al., 2011). The ExPASy server (http://us.expasy.org/tools/protparam.html) was used to predict molecular weight and isoelectric point. Prediction of transmembrane helices was performed using TMHMM 2.0 (Krogh et al., 2001) and signal peptides were screened using SignalP (Petersen et al., 2011). Potential tRNA genes were identified using tRNAscan-SE (Lowe, 1997) and ARAGON (Laslett and Canback, 2004).

The comparative genomic analysis between our bacteriophages and those deposited in GenBank was performed using BLASTn and BLAST Ring Image Generator (BRIG) (Alikhan et al., 2011). The newest proposal of International Committee on Taxonomy of Viruses (ICTV) (https://talk.ictvonline.org/files/ictv_official_taxonomy_updates_since_the_8th_report/m/prokaryote-official/5901) (Adams et al., 2017) was taken as the reference to determine the taxonomy of the bacteriophages. Mauve (http://asap.ahabs.wisc.edu/mauve/) and CoreGenes (Zafar et al., 2002; Mahadevan et al., 2009a,b) were used to compare the genomic organization and core genes for a given group of bacteriophages. Phylogenetic tree for taxonomic analysis was constructed via phylogeny.fr with “One Click mode” using MUSCLE for multiple alignments, PhyML for phylogeny, and Gblocks for eliminating poorly aligned positions and divergent regions (Anisimova and Gascuel, 2006).

**Nucleotide accession no**. Genome sequences of bacteriophages WCHABP1 and WCHABP12 in the present study have been deposited into GenBank under accession no. KY829116 and KY670595.

### Analysis of the genome ends

Genome ends were determined as described previously (Casjens and Gilcrease, 2009). Briefly, 1 μg of each bacteriophage genome DNA was digested with the restriction enzyme *Hind*III (TaKaRa, Dalian, China). The mixture was heated at 80°C for 15 min to inactivate the digestion and was divided into two equal aliquots. One was rapidly chilled in ice water bath and the other was left to slowly cool to the room temperature. The resulting DNA bands were separated in a 1% agarose gel by electrophoresis. In parallel, 1 μg of Lambda DNA standard digested with the restriction enzyme *EcoR*V (TaKaRa) was used as control.

### Sodium dodecyl sulfate–polyacrylamide gel electrophoresis (SDS-PAGE) analysis

Bacteriophage particles were boiled in loading buffer (50 mM tris-HCl, 2% Sodium dodecyl sulfate, 0.1% bromophenol blue, 10% glycerol, and 1% β-mercaptoethanol) for 5 min. Denatured proteins were then separated using sodium dodecyl sulfate–polyacrylamide gel electrophoresis (SDS-PAGE) with a 5% concentration gel and a 12% separation gel and were stained with Coomassie Blue G-250 as described previously (Boulanger, 2009).

### *Galleria mellonella* bacteriophage therapy assay

*G. mellonella* infected by *A. baumannii* was used to study *in vivo* antibacterial efficacy of our bacteriophages (Peleg and Jara, 2009). Larvae of *G. mellonella* of 250–350 mg in weight (Huiyude, Tianjin, China) were stored at 4°C and were used within 1 week after delivery. Briefly, 16 randomly chosen larvae were used for each group. Host bacterial cultures of strain Ab1186 and Ab1262, from which the two bacteriophages were recovered, were washed by phosphate-buffered saline (PBS; Beyotime, Shanghai, China) and were then diluted 1:10 to an appropriate cell density (10^7^ cfu/ml) as determined using a McFarland turbidimetry and direct plate colony counting. Aliquots of 10 μl PBS-washed bacterial cultures (10^5^ cfu) were injected into the hemocoel of each larva through the last left proleg using a microsyringe (Gaoge, Shanghai, China), which led to a pre-determined LD_80_ dose for each inoculum (10^5^ cfu/larva). Bacteriophage stocks were re-concentrated as described above and were re-suspended and diluted with PBS to the optimal MOI titer (for host cell at 10^5^ cfu/ml, the bacteriophage dosage was 10^4^ pfu/ml to reach a final MOI of 0.1 pfu/cfu). Aliquots of 10 μl bacteriophage suspensions (10^4^ pfu/larva) were then injected into the larva via a different proleg within 30 min from the bacterial inoculation. In parallel, larvae injected with bacterial cells at the same concentration were treated with polymyxin B (Meilun, Dalian, China) at 2.5 mg/kg (Hornsey and Wareham, 2011) to compare the therapeutic efficacy between antibiotics and bacteriophages. For each bacteriophage, four control groups were set up including larvae infected with bacterial cells and treated with PBS solution to observe the bacterial virulence (group 1), those injected with either the bacteriophage suspension or polymyxin B to assess their potential toxicity (group 2 and 3); those injected with PBS only to observe the potential physical trauma from injection (group 4). Larvae were then incubated in plastic containers at 37°C and the number of dead, determining as no movement under touch, was counted at 24 h intervals up to 96 h after the incubation. When more than two larvae died in any negative control (group 2, 3, and 4) at the end of observation, the assay was considered invalid and was repeated. Assays were performed in triplicate using different batches of larvae.

The statistical analysis was performed by GraphPad Prism v.7.0 (Software Inc., La Jolla, CA, USA) to plot the survival curves with the Kaplan-Meier method following a log-rank test to calculate the differences in survival. A *P*-value of < 0.05 was considered to be statistically significant.

## Results

### Two bacteriophages of the *Myoviridae* family were recovered

Two bacteriophages, designated WCHABP1 and WCHABP12, were obtained from hospital sewage with the ability to infect 9 and 12 out of the 18 host strains, exhibiting that both bacteriophages can infect multiple strains of *A. baumannii* with varied efficiency (Table 1). Upon infecting their original host strain, clear plaques of 8–9 and 5–7 mm in diameter with all surrounded by a 1–3 mm halo were formed by bacteriophage WCHABP1 and WCHABP12, respectively.

**Figure 1:**
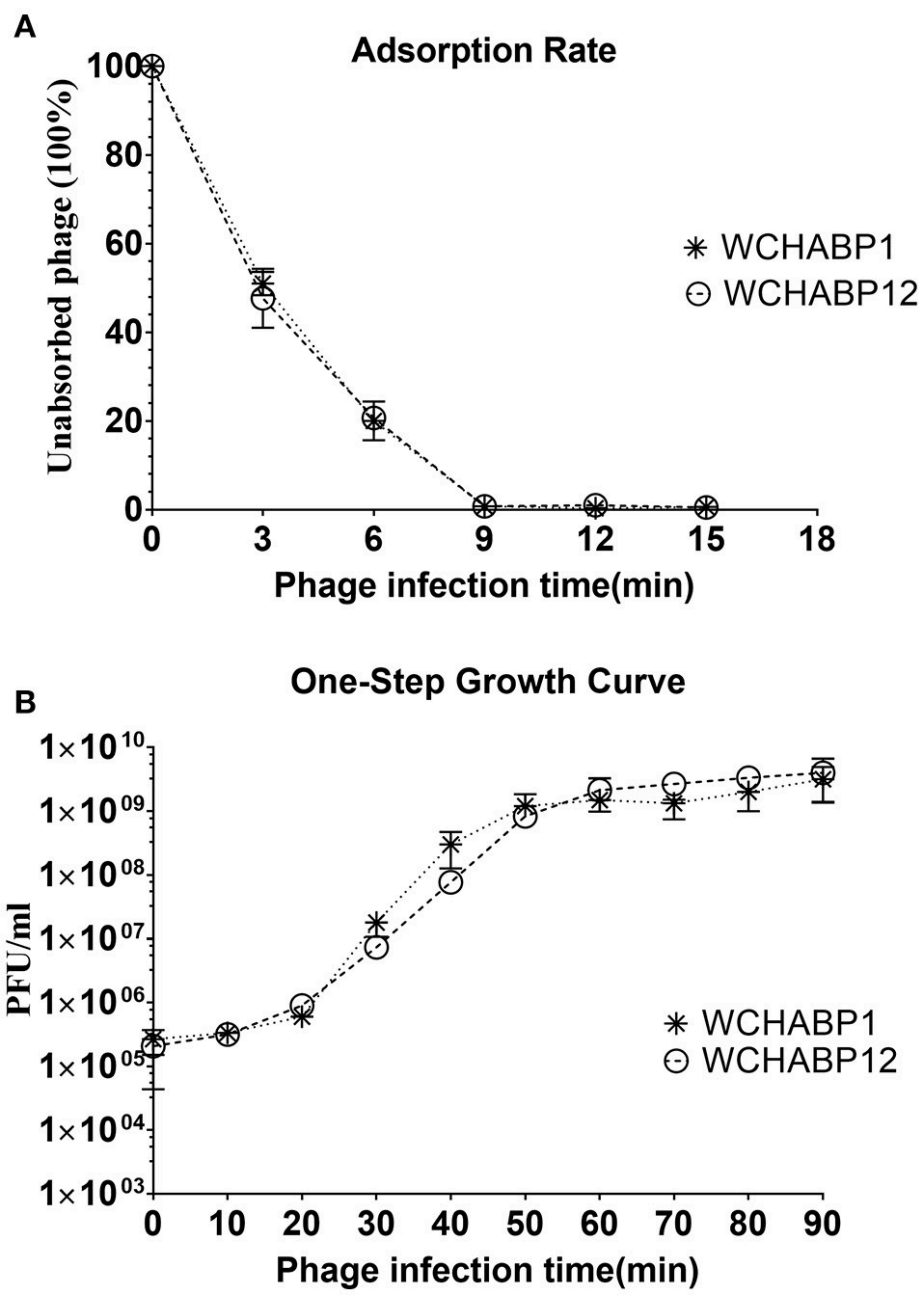
Growth and lytic characteristics. **(A)** adsorption rate; **(B)** One-step growth curve. WCHABP1 and WCHABP12 are labeled by asterisk and circle, respectively.

Both bacteriophages produced the highest progeny at MOI of 0.1 (1.55 × 10^10^ pfu/ml for WCHABP1 and 2.2 × 10^10^ for WCHABP12), which was therefore used in subsequent experiments. The adsorption rate of them was comparable where more than 99% of bacteriophage particles could adsorb onto the host cell within 10 min (Figure 1). After the adsorption, for both WCHABP1 and WCHABP12, the eclipse period was between 10 and 20 min and the plateau was reached at 60 min with a slightly different final titer (1.5 × 10^9^ pfu/ml for WCHABP1 and 2.1 × 10^9^ pfu/ml for WCHABP1; Figure 1). Considering that the initial number of infected cells were 1.1 × 10^7^ cfu/ml for WCHABP1 and 1.2 × 10^7^ cfu/ml for WCHABP12, the burst size of the two bacteriophages was 136 and 175 pfu per infected cell, respectively.

**Figure 2:**
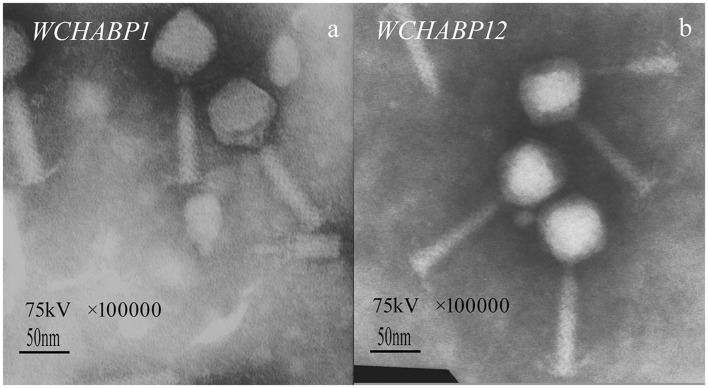
Electron microscopy of bacteriophage morphology. **(a)** WCHABP1; **(b)** WCHABP12. The black scale bar represents 50 nm.

The electron microscopy revealed that both WCHABP1 and WCHABP12 had a ~75 nm icosahedral head and a ~105 nm tail, on which crossed striation could be seen (Figure 2). The morphologic features suggested that both WCHABP1 and WCHABP12 belong to the *Myoviridae* family.

### The two bacteriophages represent two new species of the *Ap22virus* genus within the *Myoviridae* family

Genomes of the WCHABP1 and WCHABP12 were 45,888 and 45,415 bp in length with the same GC content of 37.6 mol%. The number of ORFs predicted was 88 for WCHABP1 and 89 for WCHABP12, respectively. Genomes of WCHABP1 and WCHABP12 were highly similar with each other (88.0% overall DNA sequence identity) and had 51.0 to 56.0% overall DNA sequence identity to a set of bacteriophages (Table 2) belonging to the *Ap22virus* genus of the *Myoviridae* family.

**Table 2:** Genomic properties of WCHABP1 and WCHABP12 compared with other bacteriophages of the *Ap22virus* genus.

**Bacteriophage**	**GenBank accession no**.	**Genome length (kb)**	**GC content (%)**	Overall DNA sequence identity (%)^*^	Homologous proteins (%)^*^	**No. of CDS**	**No. of tRNAs**
AP22	HE806280	46.3	37.7	–	–	89	0
IME-AB2	JX976549	46.6	37.5	49.0	64.0	82	0
AB1	HM368260	45.1	37.7	56.0	61.8	85	0
YMC-13-01-C62	KJ817802	44.8	37.6	53.0	65.4	84	0
WCHABP1	KY829116	45.8	37.6	58.0	74.2	89	0
WCHABP12	KY670595	45.4	37.6	62.0	75.3	88	0

* Bacteriophage AP22 was used as the reference for determining overall DNA sequence identity and homologous proteins

To investigate the taxonomy of WCHABP1 and WCHABP12, we followed the approach of the recently approved taxonomic proposal for the *Ap22virus* genus (Adams et al., 2017). Bacteriophages of this genus possess genome of ~45 kb (around 37.6 mol% G+C content) and encode ORFs close to 85 in number with no tRNAs. Members of the *Ap22virus* genus had an average of 53% overall DNA sequence identity and 64% homologous proteins compared with the reference species bacteriophage AP22. WCHABP1 and WCHABP12 exhibited a higher overall DNA sequence identity (58 and 62%) and homologous proteins (74 and 75%) with AP22, carrying almost the same number of ORFs as AP22 (88 and 89 vs. 89). The multiple genome alignment of WCHABP1, WCHABP12, and bacteriophages of the *Ap22virus* genus showed that all of these genomes were in a resembling gene cluster and order (Figure S1). Phylogenetic trees constructed with bacteriophage major capsid and baseplate J-like protein revealed that both WCHABP1 and WCHABP12 were clustered together within the *Ap22virus* genus (Figure S2). All above findings suggest that WCHABP1 and WCHABP12 belonged to the *Ap22virus* genus. In addition, WCHABP1 and WCHABP12 had <95.0% identity between each other and with any other bacteriophages of the *Ap22virus* genus. Therefore, WCHABP1 and WCHABP12 present two new species of the *Ap22virus* genus.

### Genomes of the two bacteriophages encode proteins for morphogenesis, genome packaging, nucleotide metabolism, and host lysis

**Figure 3:**
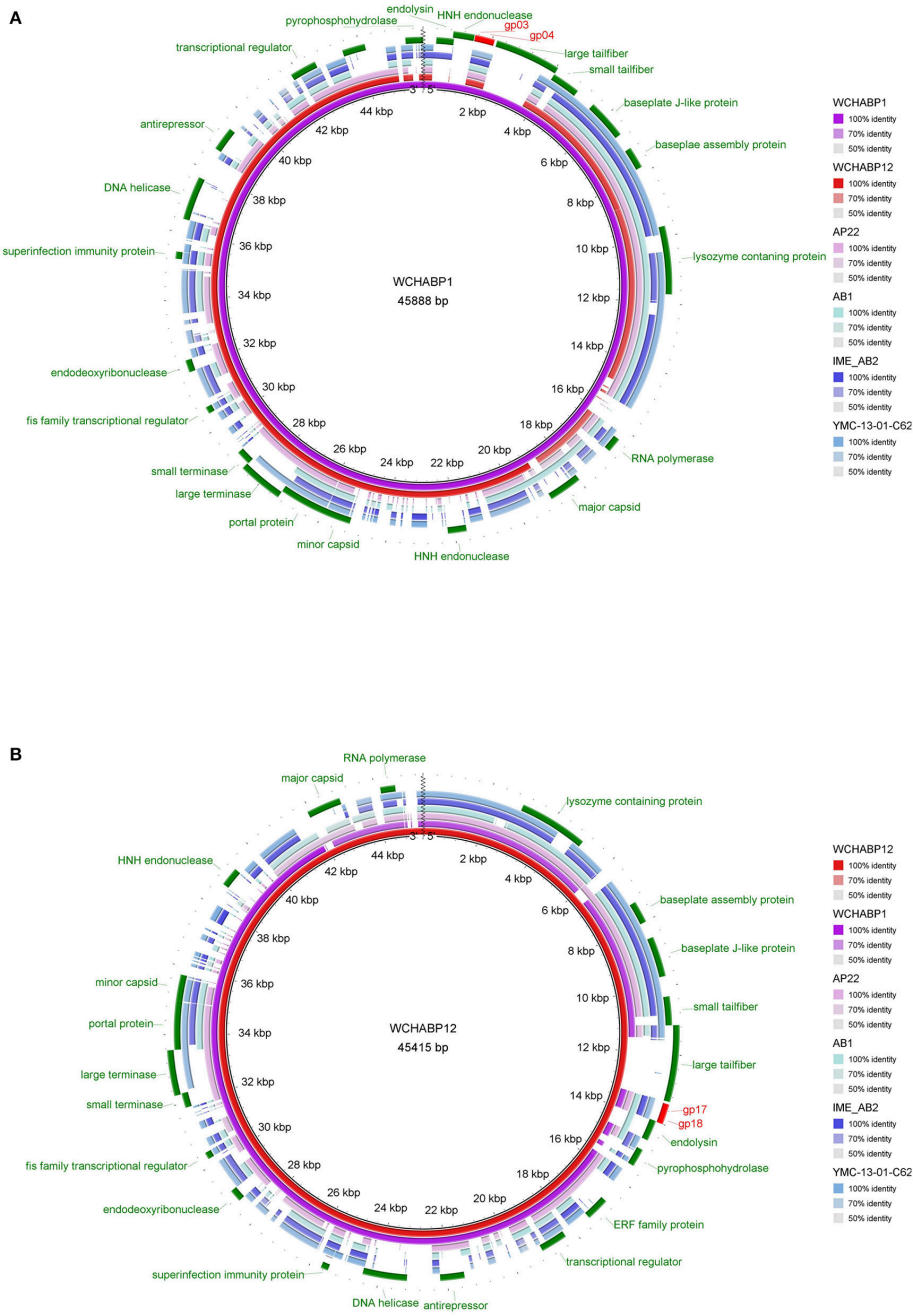
Multiple genome alignments of WCHABP1, WCHABP12, and bacteriophages of the *Ap22virus* genus. **(A)** the genome of WCHABP1 is used as the reference. **(B)** the genome of WCHABP12 is used as the reference. The alignment is a pairwise BLASTn performed using BRIG. Accession numbers of genomes of bacteriophages of the *Ap22virus* genus are listed in Table 2.

Among ORFs of WCHABP1 and WCHABP1, 22 (WCHABP1) and 21 (WCHABP12) could be assigned to known function including morphogenesis, genome packaging, nucleotide metabolism, and host lysis. Neither tRNAs nor genes involving in the lysogenic process such as integrase and repressor were identified in both bacteriophages (Table 2, Figure 3).

#### Morphology module

The SDS-PAGE analysis showed similar protein patterns between WCHABP1 and WCHABP12 (Figure S3). The protein bands mainly converged at 15, 35, 40–55, and 70 kDa, corresponding to the respective capsid, portal and tail fiber proteins of the two bacteriophages in light of the predicted molecular weight.

Bioinformatics analysis predicted seven ORFs encoding structural proteins for the two bacteriophages. These structural proteins included two capsid proteins (gp27 and gp43 of WCHABP1; gp66 and gp82 of WCHABP12), one portal protein (gp44 of WCHABP1; gp65 of WCHABP12), two baseplate proteins (gp08 and gp10 of WCHABP1; gp11 and gp13 of WCHABP12), and two tail fiber proteins (gp05 and gp06 of WCHABP1; gp15 and gp16 of WCHABP12). The structural proteins encoded by each of the two bacteriophage genomes are highly similar (>60% overall amino acids identity, a detailed list is shown in Table 3) with the exception of the large tail fiber subunits (gp05 of WCHABP1 and gp16 of WCHABP12). The N-terminal 149 amino acids of the large tail fiber subunit of WCHABP1 and WCHABP12 were of 100% coverage and 76% identity, while the remaining C-terminal amino acids had no significant similarity.

**Table 3:** Genes with known function in bacteriophage WCHABP1 and WCHABP12.

**WCHABP1**			**Predicated function**	**WCHABP12**			**Coverage, identity (%, amino acid sequence)**
**ORFs**	**Location**	**Length (bp)**		**ORFs**	**Location**	**Length (bp)**	
gp05	2148–4052	1905	Large tail fiber subunit	gp16	11145–13343	2199	41, 76
gp06	4054–4899	846	Small tail fiber subunit	gp15	10298–11143	846	100, 95
gp08	5503–6687	1185	Baseplate J-like protein	gp13	8510–9694	1185	100, 99
gp10	7183–7827	645	Baseplate assembly protein	gp11	7367–8014	648	100, 94
gp14	9691–11721	2031	Lysozyme domain containing protein	gp06	3007–5037	2031	100, 94
gp23	16332–16769	438	RNA polymerase	gp86	44161–44598	438	100, 93
gp27	17988–18980	993	Major capsid	gp82	41925–42932	1008	98, 60
gp33	21655–22212	558	HNH endonuclease	gp76	38683–39240	558	100, 100
gp43	25134–25904	771	Minor capsid	gp66	34991–35761	771	100, 99
gp44	25907–27337	1431	Portal protein	gp65	33558–34988	1431	100, 100
gp45	27340–28641	1302	Large terminase subunit	gp64	32254–33555	1302	100, 100
gp46	28622–29053	432	Small terminase subunit	gp63	31842–32273	432	100, 100
gp52	30486–30686	201	Fis family transcriptional regulator	gp57	30209–30409	201	100, 100
gp56	31829–32191	363	Endodeoxyribonuclease	gp53	28704–29066	363	100, 100
gp64	35219–35431	213	Superinfection immunity protein	gp45	25464–25676	213	100, 100
gp69	36413–37714	1302	DNA helicase	gp40	23181–24482	1302	100, 100
gp72	38691–39404	714	Antirepressor protein	gp37	21491–22204	714	100, 100
gp80	41808–42599	792	Transcriptional regulator	gp29	18296–19087	792	100, 100
gp84	43473–44153	681	ERF family protein	gp25	16742–17422	681	100, 100
gp89	45367–45888	522	Nucleoside triphosphate pyrophosphohydrolase	gp21	14909–15442	534	97, 63
gp01	396–911	501	Endolysin	gp19	13985–14593	609	–
gp02	873–1484	612	HNH endonuclease				–

In addition, both WCHABP1 and WCHABP12 had a gene encoding a lysozyme-containing protein, which is also possessed by other bacteriophages of the *Ap22virus* genus. The lysozyme is a glycoside hydrolase that catalyzes the peptidoglycan of the bacterial cell wall (Strynadka and James, 1996). The lysozyme-containing protein lies close to the baseplate protein and tail fiber proteins, suggesting that it might be a part of tail. Lysozyme could be released from the inner tail tube to create a small hole in the cell wall peptidoglycan layer that allows the double-stranded DNA (dsDNA) genome to enter the cell (Duda, 2008). Nonetheless, the exact function of the lysozyme-containing protein in the bacteriophages warrants further studies.

#### Packaging module and genome termini

Two ORFs encoding terminase, which is the key component of the packaging machine, were identified in both bacteriophage genomes (gp45 and gp46 of WCHABP1; gp63 and gp64 of WCHABP12). Terminase for bacteriophages usually comprises two subunits, where the large subunit translocates bacteriophage DNA into empty capsids and cuts it at a unique and precise dsDNA sequence to accomplish the packaging process, while the small subunit contributes to binding of the packaging initiation site and regulates the ATPase activity for the large subunit (Feiss and Rao, 2012). Amino acid sequences of terminase subunits between WCHABP1 and WCHABP12 were identical (Table 3). The restriction map of genomes of WCHABP1 and WCHABP12 showed no change in band patterns after endonuclease restriction with rapid or slow cooling, suggesting the absence of cohesive ends in their genomes (Figure S4).

#### Nucleotide metabolism module

Both WCHABP1 and WCHABP12 had 10 genes involving in genome replication, transcription, establishment of infection, and other processes, including a RNA polymerase, a DNA helicase, a transcriptional regulator, a *fis* family transcriptional regulator, an ERF (essential recombination function) family protein, a nucleoside triphosphate pyrophosphohydrolase, a HNH endonuclease, an endodeoxyribonuclease, a superinfection immunity protein and a bacteriophage antirepressor protein (Table 2). Products of these genes were highly similar (93–100% overall amino acids identity) between the two bacteriophages except for the nucleotide triphosphate pyrophosphohydrolase (gp89 of WCHABP1 and gp21 of WCHABP12) with a 97% coverage but only 63% amino acid identity. Both gp89 of WCHABP1 and gp21 of WCHABP12 contained an NTP-PPase_u3 domain (also known as MazG domain, CCD accession cd11540), suggesting a similar function. Previous study suggests that the MazG domain-containing proteins might contribute to extending the logarithmic phase of bacterial growth, facilitating the production of bacteriophage progeny via the reactivation of metabolic pathways that are usually suppressed under nutrient starvation (Bryan et al., 2008).

Besides the identical HNH endonuclease seen in both WCHABP1 (gp33) and WCHABP12 (gp76), WCHABP1 had another HNH endonuclease (gp02), which is absent from WCHABP12 and any other members of the *Ap22virus* genus and has a relatively low similarity (31% coverage and 31% identity) to the aforementioned HNH endonuclease of WCHABP1 and WCHABP12. The gp02 product of WCHABP1 consisted of a HNH endonuclease domain (Pfam accession PF13392.5) and an AP2 domain (Pfam accession PF00847.19), while the gp33 product of WCHABP1 and gp76 product of WCHABP12 contained a HNH endonuclease domain (Pfam accession PF13392.5) and a NUMOD4 motif (Pfam accession PF07463.10). The HNH endonuclease domain is involved in the process of homing, while both AP2 domain and NUMOD4 motif play a role in DNA binding (Ohmetakagi and Shinshi, 1995; Sitbon and Pietrokovski, 2003) but without significant amino acid similarity.

#### Lysis control

WCHABP1 and WCHABP12 had one gene encoding the endolysin. However, endolysins of the two bacteriophages had no significant similarity in amino acid sequences. The endolysin of WCHABP1 (gp01) had 171 amino acids and contains a glycosyl hydrolase 108 family domain (Pfam accession PF05838.11) with catalytic activity as a N-acetylmuramidase (Stojković and Rothman-Denes, 2007) and a peptidoglycan domain for substrate binding (Pfam accession PF09374.9). By contrast, the endolysin of WCHABP12 (gp19) had 202 amino acids with a single domain belonging to the glycoside hydrolase family 19 (Pfam accession PF00182.18), which is able to hydrolyze the β-1,4-N-acetyl-D-glucosamine linkages in chitin polymers and leads to the breakage of chitin-containing cell walls (Eijsink et al., 2008). The different types of endolysins between WCHABP1 and WCHABP12 suggest that even closely-related bacteriophages may target different substrates of the host cell wall.

Most dsDNA bacteriophages with a genome size larger than 10 kb employ a complete set of a holin-endolysin system to release the phage progeny (Young and White, 2008). The holin first permeabilizes the host cell membrane and endolysin can therefore reach the periplasm to break down the peptidoglycan layer (Young et al., 2000). However, for both WCHABP1 and WCHABP12 no known holin-encoding genes were identified using both a BLASTp search and domain/motif prediction.

### *In vivo* therapeutic efficacy

**Figure 4:**
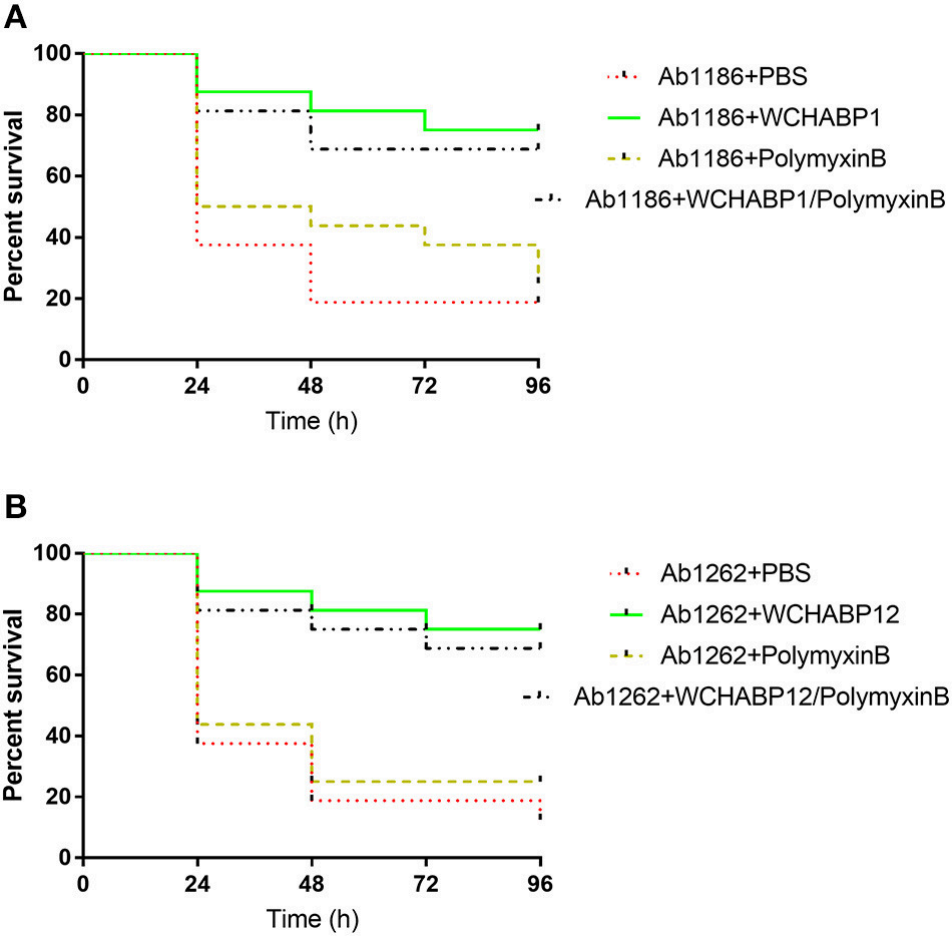
Survival of *G. mellonella* larvae after infection by carbapenem-resistant *A. baumannii* clinical strains. **(A)** WCHABP1; **(B)** WCHABP12. Ab1186 and Ab1262 are two carbapenem-resistant *A. baumannii* clinical strains, from which bacteriophages WCHABP1, WCHABP12 were recovered. The detailed survival rates are listed in Table S2.

For larvae infected by carbapenem-resistant *A. baumannii* strains, the treatment of WCHABP1 and WCHABP12 significantly improved the survival rates (Figure 4 and Table S2). For those infected by strain Ab1186, survival of larvae at 96 h was 75.00 and 18.75% (*p* < 0.01) with and without the treatment of WCHABP1, respectively. Similarly, for those infected by strain Ab1262, survival was 75 and 12.5% (*p* < 0.01) with and without the treatment of WCHABP12, respectively. By contrast, the treatment of polymyxin B only slightly increased the survival, which is not statistically significant. With and without the treatment of polymyxin B, the survival rate of those infected by strain Ab1186 was 25 and 18.75% (*p* = 0.46), respectively, and that of those infected by Ab1262 was 25 and 12.5% (*p* = 0.47), respectively. The treatment of the combination of bacteriophage and polymyxin B did not further enhance the survival of larvae infected by strain Ab1186 or Ab1262 compared with the treatment of bacteriophage alone (68.75 vs. 75%, *p* = 0.687).

## Discussion

The recent success in treating the infection caused by MDR *A. baumannii* by topical and systematic administration of varied bacteriophages together with antibiotics once again highlights the potential of bacteriophages as promising alternatives against bacterial infections that are difficult to be treated otherwise (https://www.sciencedaily.com/releases/2017/04/170425124826.htm). Nonetheless, constant works on isolation and characterization of new bacteriophages as well as their *in vitro* and *in vivo* test for both safety and therapeutic efficacy are still imperative. In this study, we reported two bacteriophages, WCHABP1 and WCHABP12, belonging to the *Ap22virus* genus of the *Myoviridae* family with a large burst size and short lytic life cycle against the carbapenem-resistant *A. baumannii* host strains. The bacteriophages identified in the present study together with others discovered in previous studies (Lee et al., 2011; Jin et al., 2012; Popova et al., 2012; Ghajavand et al., 2017) may expand our arsenal against *A. baumannii*.

The injection of bacteriophages WCHABP1 and WCHABP12 did not cause any death of larvae, suggesting that they were not toxic for the larvae. The administration of WCHABP1 and WCHABP12 was able to rescue most larvae infected by carbapenem-resistant *A. baumannii* susceptible to the lysis of the bacteriophages, suggesting both bacteriophages new potent weapons against *A. baumannii*. By contrast, the treatment of polymyxin B alone was only able to rescue few larvae infected by these strains that were susceptible to the antibiotic, which is consistent with the findings of another study (Hornsey et al., 2013). A few factors may contribute to the failure of polymyxin B treatment such as emerging tolerance and resistance to the antibiotic after exposure (Li et al., 2006; Harding et al., 2018).

For products encoded by the genomes of WCHABP1, WCHABP12, and other members of the *Ap22virus* genus, there are several noteworthy features. First, the C-terminal amino acid sequence of the large tail fiber subunit had no significant similarity between WCHABP1 and WCHABP12. Such highly variable C-terminal sequences of large tail fiber subunits are also present among other members of the *Ap22virus* genus. Previous studies have revealed that the large tail fiber subunit of various bacteriophages including those belonging to the *Myoviridae* family has a conserved N-terminus and a variable C-terminus, which determines the host specificity by binding the receptor on the surface of host cell (Rakhuba et al., 2010; Kurtböke, 2012). The variable C-terminal sequence of large tail fiber subunits may therefore account for the different host ranges of bacteriophages of the *Ap22virus* genus. Second, like those of other bacteriophages of the *Ap22virus* genus, the large terminase subunit of WCHABP1 and WCHABP12 had a terminase_3 family domain (Pfam accession pfam04466) and were distantly homologous (26% identity with a E-value of 2e-22) with that of bacteriophage SPP1 (GenBank accession NC_004166), which is a well-studied model system for headful DNA packaging mechanism (also known as *pac*-mechanism; Oliveira et al., 2013). This suggests that both WCHABP1 and WCHABP12 also employ a *pac*-mechanism for genome packaging and their genomes are circularly permuted and terminally redundant (Casjens and Gilcrease, 2009; Oliveira et al., 2013). Third, surprisingly, unlike the typical holin-endolysin set seen in most bacteriophages, no known holin-encoding genes were identified for bacteriophages of the *Ap22virus* genus. Two ORFs (gp3 and gp4 of WCHABP1; gp17 and gp18 of WCHABP12) were present between genes encoding endolysin and the large tail fiber subunit and were highly similar among members of the *Ap22virus* genus. The products encoded by the two ORFs had two and three transmembrane domains as identified using TMHMM. As most holins contain transmembrane domains (Young et al., 2000) and their encoding genes usually locate proximately to endolysin-encoding genes, it is reasonable to hypothesize that the two ORFs may encode a new, yet-to-be-characterized holin. Alternatively, as shown in a previous study there are means other than holin for bacteriophages to permeabilize the host cell membrane via inhibiting the specific host enzymes and impairing peptidoglycan biosynthesis (Bernhardt et al., 2001). Therefore, the exact lysis mechanism employed by bacteriophages of the *Ap22virus* genus remains unclear and warrants further studies.

Although genomes of bacteriophages WCHABP1 and WCHABP12 were highly similar, there were several differences, which have been demonstrated above and are summarized here. Endolysins of WCHABP1 and WCHABP12 had no significant similarity in amino acid sequences. WCHABP1 had one more HNH endonuclease than WCHABP12. The C-terminal amino acids of the large unit of tail fiber protein between the two bacteriophages had no significant similarity. In addition, WCHABP1 had three genes without known function that were absent from WCHABP12, while two genes with unknown function of WCHABP12 were not present in WCHABP1.

In conclusion, two new bacteriophages, exhibiting activity to infect and kill multiple carbapenem-resistant *A. baumannii* clinical strains, were recovered. The two bacteriophages represent two new species of the *Ap22virus* genus within the *Myoviridae* family. Administration of the two bacteriophages was effective to treat carbapenem-resistant *A. baumannii* infections in the *G. mellonella* larvae model. The findings could expand our sight on *Acinetobacter* bacteriophages and offer new potential therapeutic alternatives.

## Author contributions

ZZ designed the study. WZ performed the experiments and prepared figures. WZ, YF, and ZZ analyzed and interpreted the data. WZ and ZZ wrote the manuscript.

### Conflict of interest statement

The authors declare that the research was conducted in the absence of any commercial or financial relationships that could be construed as a potential conflict of interest.

## References

[B1] Abubakar S. Suleiman B. H. Abbagana B. A. Mustafa I. A. Musa I. A. (2016). Novel uses of bacteriophages in the treatment of human infections and antibiotic resistance. Am. J. Biosci. 4, 34–40. 10.11648/j.ajbio.20160403.13

[B2] Adams M. J. Lefkowitz E. J. King A. M. Q. Harrach B. Harrison R. L. Knowles N. J.. (2017). Changes to taxonomy and the international code of virus classification and nomenclature ratified by the international committee on taxonomy of viruses (2017). Arch. Virol. 162, 1–34. 10.1007/s00705-017-3358-5 28434098

[B3] Alikhan N. F. Petty N. K. Ben Zakour N. L. Beatson S. A. (2011). BLAST Ring Image Generator (BRIG): simple prokaryote genome comparisons. BMC Genomics 12:402. 10.1186/1471-2164-12-402 21824423 PMC3163573

[B4] Altschul S. F. Gish W. Miller W. Myers E. W. Lipman D. J. (1990). Basic local alignment search tool. J. Mol. Biol. 215, 403–410. 10.1016/S0022-2836(05)80360-2 2231712

[B5] Altschul S. F. Madden T. L. Schäffer A. A. Zhang J. Zhang Z. Miller W.. (1997). Gapped BLAST and PSI-BLAST: a new generation of protein database search programs. Nucleic Acids Res. 25, 3389–3402. 10.1093/nar/25.17.3389 9254694 PMC146917

[B6] Anisimova M. Gascuel O. (2006). Approximate likelihood-ratio test for branches: a fast, accurate, and powerful alternative. Syst. Biol. 55, 539–552. 10.1080/10635150600755453 16785212

[B7] Bankevich A. Nurk S. Antipov D. Gurevich A. A. Dvorkin M. Kulikov A. S.. (2012). SPAdes: a new genome assembly algorithm and its applications to single-cell sequencing. J. Comput. Biol. 19, 455–477. 10.1089/cmb.2012.0021 22506599 PMC3342519

[B8] Berlau J. Aucken H. M. Houang E. Pitt T. L. (1999). Isolation of *Acinetobacter* spp. including *A. baumannii* from vegetables: implications for hospital-acquired infections. J. Hosp. Infect. 42, 201–204. 10.1053/jhin.1999.0602 10439992

[B9] Bernhardt T. G. Wang I. N. Struck D. K. Young R. (2001). A protein antibiotic in the phage Qβ virion: diversity in lysis targets. Science 292, 2326–2329. 10.1126/science.1058289 11423662

[B10] Boulanger P. (2009). Purification of bacteriophages and SDS-PAGE analysis of phage structural proteins from ghost particles. Methods Mol. Biol. 502, 227. 10.1007/978-1-60327-565-1_13 19082559

[B11] Bruynoghe R. Maisin J. (1921). Essais de thérapeutique au moyen du bacteriophage. CR Soc. Biol. 85, 1120–1121.

[B12] Bryan M. J. Burroughs N. J. Spence E. M. Clokie M. R. J. Mann N. H. Bryan S. J. (2008). Evidence for the intense exchange of mazG in marine cyanophages by horizontal gene transfer. PLoS ONE 3:e2048. 10.1371/journal.pone.0002048 18431505 PMC2297514

[B13] Casjens S. R. Gilcrease E. B. (2009). Determining DNA packaging strategy by analysis of the termini of the chromosomes in tailed-bacteriophage virions. Methods Mol. Biol. 502, 91–111. 10.1007/978-1-60327-565-1_7 19082553 PMC3082370

[B14] CLSI (2017). Performance Standards for Antimicrobial Susceptibility Testing; Twenty-Third Informational Supplement. M100-S27. (Wayne, PA: Clinical and Laboratory Standards Institute).

[B15] d'herelle F. (1917). Sur un microbe invisible antagoniste des bacilles dysentériques. CR Acad. Sci. Paris 165, 373–375.

[B16] Di P. A. Giannouli M. Triassi M. Brisse S. Zarrilli R. (2011). Molecular epidemiological investigation of multidrug-resistant *Acinetobacter baumannii* strains in four Mediterranean countries with a multilocus sequence typing scheme. Clin. Microb. Infect. 17, 197–201. 10.1111/j.1469-0691.2010.03254.x 20456455

[B17] Dijkshoorn L. Nemec A. Seifert H. (2007). An increasing threat in hospitals: multidrug-resistant *Acinetobacter baumannii*. Nat. Rev. Microbiol. 5, 939–951. 10.1038/nrmicro1789 18007677

[B18] Duda R. L. (2008). Icosahedral tailed dsDNA bacterial viruses. Encycl. Virol. 30–37. 10.1016/B978-012374410-4.00754-8

[B19] Eijsink V. G. H. Vaaje-Kolstad G. Vårum K. M. Horn S. J. (2008). Towards new enzymes for biofuels: lessons from chitinase research. Trends Biotechnol. 26, 228–235. 10.1016/j.tibtech.2008.02.004 18367275

[B20] Feiss M. Rao V. B. (2012). The bacteriophage DNA packaging machine. Adv. Exp. Med. Biol. 726, 489–509. 10.1007/978-1-4614-0980-9_22 22297528

[B21] Ghajavand H. Esfahani B. N. Havaei A. Fazeli H. Jafari R. Moghim S. (2017). Isolation of bacteriophages against multidrug resistant *Acinetobacter baumannii*. Res. Pharm. Sci. 12, 373–380. 10.4103/1735-5362.213982 28974975 PMC5615867

[B22] Harding C. M. Hennon S. W. Feldman M. F. (2018). Uncovering the mechanisms of *Acinetobacter baumannii* virulence. Nat. Rev. Microbiol. 16, 91–102. 10.1038/nrmicro.2017.148 29249812 PMC6571207

[B23] Helvoort T. V. (2001). Felix d'Herelle and the origins of molecular biology. J. Hist. Med. Allied Sci. 56, 305–307. 10.1093/jhmas/56.3.305

[B24] Hornsey M. Wareham D. W. (2011). *In vivo* efficacy of glycopeptide-colistin combination therapies in a *Galleria mellonella* model of *Acinetobacter baumannii* infection. Antimicrob. Agents Chemother. 55, 3534–3537. 10.1128/AAC.00230-11 21502628 PMC3122470

[B25] Hornsey M. Phee L. Longshaw C. Wareham D. W. (2013). *In vivo* efficacy of telavancin/colistin combination therapy in a *Galleria mellonella* model of *Acinetobacter baumannii* infection. Int. J. Antimicrob. Agents 41, 285–287. 10.1016/j.ijantimicag.2012.11.013 23312607

[B26] Jin J. Li Z. J. Wang S. W. Wang S. M. Huang D. H. Li Y. H.. (2012). Isolation and characterization of ZZ1, a novel lytic phage that infects *Acinetobacter baumannii* clinical isolates. BMC Microbiol. 12:156. 10.1186/1471-2180-12-156 22838726 PMC3438129

[B27] Khan M. M. Nilsson A. S. (2015). Isolation of phages for phage therapy: a comparison of spot tests and efficiency of plating analyses for determination of host range and efficacy. PLoS ONE 10:e0118557 10.1371/journal.pone.0118557 25761060 PMC4356574

[B28] Krawczyk B. Lewandowski K. Kur J. (2002). Comparative studies of the Acinetobacter genus and the species identification method based on the recA sequences. Mol. Cell. Probes 16, 1–11. 10.1006/mcpr.2001.0388 12005442

[B29] Krogh A. Larsson B. Von Heijne G. Sonnhammer E. L. (2001). Predicting transmembrane protein topology with a hidden Markov model: application to complete genomes. J. Mol. Biol. 305, 567–580. 10.1006/jmbi.2000.4315 11152613

[B30] Kropinski A. M. Mazzocco A. Waddell T. E. Lingohr E. Johnson R. P. (2009). Enumeration of bacteriophages by double agar overlay plaque assay. Methods Mol. Biol. 501, 69–76. 10.1007/978-1-60327-164-6_7 19066811

[B31] Kurtböke I. (2012). Bacteriophages. Rijeka: InTech 10.5772/1065

[B32] Kutter E. M. Kuhl S. J. Abedon S. T. (2015). Re-establishing a place for phage therapy in western medicine. Future Microbiol. 10, 685–688. 10.2217/fmb.15.28 26000644

[B33] Laslett D. Canback B. (2004). ARAGORN, a program to detect tRNA genes and tmRNA genes in nucleotide sequences. Nucleic Acids Res. 32, 11–16. 10.1093/nar/gkh152 14704338 PMC373265

[B34] Lee C. N. Tseng T. T. Lin J. W. Fu Y. C. Weng S. F. Tseng Y. H. (2011). Lytic myophage Abp53 encodes several proteins similar to those encoded by host *Acinetobacter baumannii* and phage phiKO2. Appl. Environ. Microbiol. 77, 6755–6762. 10.1128/AEM.05116-11 21821767 PMC3187083

[B35] Li J. Rayner C. R. Nation R. L. Owen R. J. Spelman D. Tan K. E.. (2006). Heteroresistance to colistin in multidrug-resistant *Acinetobacter baumannii*. Antimicrob. Agents Chemother. 50, 2946–2950. 10.1128/AAC.00103-06 16940086 PMC1563544

[B36] Lowe T. (1997). tRNAscan-SE: a program for improved transfer RNA detection in genomic sequence (release: 1.23). Nucleic Acids Res. 25, 955–964. 10.1093/nar/25.5.0955 9023104 PMC146525

[B37] Mahadevan P. King J. F. Seto D. (2009a). CGUG: *in silico* proteome and genome parsing tool for the determination of “core” and unique genes in the analysis of genomes up to ca. 1.9 Mb. BMC Res. Notes 2:168. 10.1186/1756-0500-2-168 19706165 PMC2738686

[B38] Mahadevan P. King J. F. Seto D. (2009b). Data mining pathogen genomes using geneorder and coregenes and CGUG: gene order, synteny and *in silico* proteomes. Int. J. Comput. Biol. Drug Des. 2, 100–114. 10.1504/IJCBDD.2009.027586 20054988

[B39] Mandell J. D. Hershey A. D. (1960). A fractionating column for analysis of nucleic acids. Anal. Biochem. 1, 66–77. 10.1016/0003-2697(60)90020-8 14420563

[B40] Marchlerbauer A. Lu S. Anderson J. B. Chitsaz F. Derbyshire M. K. Deweesescott C. (2011). CDD: a conserved domain database for the functional annotation of proteins. Nucleic Acids Res. 39, D225–D229. 10.1093/nar/gkq1189 21109532 PMC3013737

[B41] Merabishvili M. Pirnay J.-P. Verbeken G. Chanishvili N. Tediashvili M. Lashkhi N.. (2009). Quality-controlled small-scale production of a well-defined bacteriophage cocktail for use in human clinical trials. PLoS ONE 4:e4944. 10.1371/journal.pone.0004944 19300511 PMC2654153

[B42] Ohmetakagi M. Shinshi H. (1995). Ethylene-inducible DNA binding proteins that interact with an ethylene-responsive element. Plant Cell 7, 173–182. 10.1105/tpc.7.2.173 7756828 PMC160773

[B43] Oliveira L. Tavares P. Alonso J. C. (2013). Headful DNA packaging: bacteriophage SPP1 as a model system. Virus Res. 173, 247–259. 10.1016/j.virusres.2013.01.021 23419885

[B44] Peleg A. Y. Jara S. D. (2009). Galleria mellonella as a model system to study *Acinetobacter baumannii* pathogenesis and therapeutics. Antimicrob. Agents Chemother. 53, 2605–2609. 10.1128/AAC.01533-08 19332683 PMC2687231

[B45] Peleg A. Y. Seifert H. Paterson D. L. (2008). *Acinetobacter baumannii*: emergence of a successful pathogen. Clin. Microbiol. Rev. 21, 538–582. 10.1128/CMR.00058-07 18625687 PMC2493088

[B46] Peng F. Mi Z. Huang Y. Yuan X. Niu W. Wang Y.. (2014). Characterization, sequencing and comparative genomic analysis of vB_AbaM-IME-AB2, a novel lytic bacteriophage that infects multidrug-resistant *Acinetobacter baumannii* clinical isolates. BMC Microbiol. 14:181. 10.1186/1471-2180-14-181 24996449 PMC4094691

[B47] Perez F. Hujer A. M. Hujer K. M. Decker B. K. Rather P. N. Bonomo R. A. (2007). Global challenge of multidrug-resistant *Acinetobacter baumannii*. Antimicrob. Agents Chemother. 51, 3471–3484. 10.1128/AAC.01464-06 17646423 PMC2043292

[B48] Perez F. Ponceterashima R. Adams M. D. Bonomo R. A. (2011). Are we closing in on an “elusive enemy”? The current status of our battle with *Acinetobacter baumannii*. Virulence 2, 86–90. 10.4161/viru.2.2.15748 21499025

[B49] Petersen T. N. Brunak S. Von H. G. Nielsen H. (2011). SignalP 4.0: discriminating signal peptides from transmembrane regions. Nat. Methods 8, 785–786. 10.1038/nmeth.1701 21959131

[B50] Popova A. V. Zhilenkov E. L. Myakinina V. P. Krasilnikova V. M. Volozhantsev N. V. (2012). Isolation and characterization of wide host range lytic bacteriophage AP22 infecting *Acinetobacter baumannii*. FEMS Microbiol. Lett. 332, 40–46. 10.1111/j.1574-6968.2012.02573.x 22506502

[B51] Quevillon E. Silventoinen V. Pillai S. Harte N. Mulder N. Apweiler R.. (2005). InterProScan: protein domains identifier. Nucleic Acids Res. 33, W116–W120. 10.1093/nar/gki442 15980438 PMC1160203

[B52] Rakhuba D. V. Kolomiets E. I. Dey E. S. Novik G. I. (2010). Bacteriophage receptors, mechanisms of phage adsorption and penetration into host cell. Pol. J. Microbiol. 59, 145–155. 10.1016/j.micres.2015.01.008.1.94 21033576

[B53] Seemann T. (2014). Prokka: rapid prokaryotic genome annotation. Bioinform 30, 2068–2069. 10.1093/bioinformatics/btu153 24642063

[B54] Sitbon E. Pietrokovski S. (2003). New types of conserved sequence domains in DNA-binding regions of homing endonucleases. Trends Biochem. Sci. 28, 473–477. 10.1016/S0968-0004(03)00170-1 13678957

[B55] Smith H. W. Huggins M. B. (1983). Effectiveness of phages in treating experimental *Escherichia coli* diarrhoea in calves, piglets and lambs. J. Gen. Microbiol. 129:2659. 10.1099/00221287-129-8-2659 6355391

[B56] Soothill J. S. (1992). Treatment of experimental infections of mice with bacteriophages. J. Med. Microbiol. 37, 258–261. 10.1099/00222615-37-4-258 1404324

[B57] Stojković E. A. Rothman-Denes L. B. (2007). Coliphage N4 N-acetylmuramidase defines a new family of murein hydrolases. J. Mol. Biol. 366, 406–419. 10.1016/j.jmb.2006.11.028 17174325

[B58] Strynadka N. C. James M. N. (1996). Lysozyme: a model enzyme in protein crystallography. EXS 75, 185–222. 10.1007/978-3-0348-9225-4_11 8765301

[B59] World Health Organization (2017). Global Priority List of Antibiotic-Resistant Bacteria to Guide Research, Discovery, and Development of New Antibiotics. Geneva: World Health Organization.

[B60] Young I. Wang I. Roof W. D. (2000). Phages will out: strategies of host cell lysis. Trends Microbiol. 8, 120–128. 10.1016/S0966-842X(00)01705-4 10707065

[B61] Young R. F. III. White R. L. (2008). Lysis of the host by bacteriophage. Encycl. Virol. 248–258. 10.1016/B978-012374410-4.00752-4

[B62] Zafar N. Mazumder R. Seto D. (2002). Coregenes: a computational tool for identifying and cataloging “core” genes in a set of small genomes. BMC Bioinform. 3:12. 10.1186/1471-2105-3-12 11972896 PMC111185

