# Supplementary material for: Two New Lytic Bacteriophages of the Myoviridae Family Against Carbapenem-Resistant Acinetobacter baumannii
Source: Front Microbiol. 2018 Apr 30;9:850. doi: 10.3389/fmicb.2018.00850 (PMC5936750; doi:10.3389/fmicb.2018.00850)
Supplement: Supplementary file 1 [file Table_1.DOCX]

**Table S1. Antimicrobial susceptibility profiles of the *A. baumannii* strains**

| Antimicrobial | Strain | | | | | | | | | | | | | | | | | |
| --- | --- | --- | --- | --- | --- | --- | --- | --- | --- | --- | --- | --- | --- | --- | --- | --- | --- | --- |
|  | Ab  1138 | Ab  1186 | Ab  1262 | Ab  1334 | Ab  1337 | Ab  1369 | Ab  1391 | Ab  1397 | Ab  1412 | Ab  1415 | Ab  1454 | Ab  1478 | Ab  1497 | Ab  1531 | Ab  1585 | Ab  1588 | Ab  1623 | Ab  1673 |
| AMC | R | R | R | R | R | R | R | R | R | R | R | R | R | R | R | R | R | R |
| AMK | S | S | S | S | S | S | R | R | S | S | S | S | S | S | S | R | S | R |
| AMP | R | R | R | R | R | R | R | R | R | R | R | R | R | R | R | R | R | R |
| ATM | R | R | R | R | R | R | R | R | R | R | R | R | R | R | R | R | R | R |
| CIP | R | R | R | R | R | R | R | R | R | R | R | R | R | R | R | R | R | R |
| COL | S | S | S | S | S | S | S | S | S | S | S | S | S | S | S | S | S | S |
| CRO | R | R | R | R | R | R | R | R | R | R | R | R | R | R | R | R | R | R |
| CSL | R | R | R | R | R | R | R | R | R | R | R | R | R | R | R | R | R | R |
| CZO | R | R | R | R | R | R | R | R | R | R | R | R | R | R | R | R | R | R |
| FEP | R | R | R | R | R | R | R | R | R | R | R | R | R | R | R | R | R | R |
| FOX | R | R | R | R | R | R | R | R | R | R | R | R | R | R | R | R | R | R |
| GEN | R | R | R | R | R | R | R | R | R | R | R | R | R | R | R | R | S | R |
| IMP | R | R | R | R | R | R | R | R | R | R | R | R | R | R | R | R | R | R |
| LVX | I | R | I | R | I | R | R | R | R | R | R | R | R | R | R | R | I | I |
| MEM | R | R | R | R | R | R | R | R | R | R | R | R | R | R | R | R | R | R |
| NIT | R | R | R | R | R | R | R | R | R | R | R | R | R | R | R | R | R | R |
| PMB | S | S | S | S | S | S | S | S | S | S | S | S | S | S | S | S | S | S |
| SAM | R | R | R | R | R | R | R | R | R | R | R | R | R | R | R | R | R | R |
| SMZ/TMP | S | S | S | S | S | S | R | R | R | R | S | R | R | R | R | R | R | R |
| TGC | S | S | S | S | S | S | S | S | S | S | S | S | S | S | S | S | S | S |
| TOB | R | R | R | R | R | R | R | R | R | R | R | R | R | R | R | R | S | R |
| TZP | R | R | R | R | R | R | R | R | R | R | R | R | R | R | R | R | R | R |
| Antibiotic abbreviation: AMC, Amoxycillin/clavulanic acid; AMK, Amikacin; AMP, Ampicillin; AZM, Aztreonam; CIP, Ciprofloxacin; COL, Colistin (Polymyxin E); CRO, Ceftriaxone; CSL, Cefoperazone/sulbactam; CZO, Cefazolin; FEP, Cefepime; FOX, Cefoxitin; GEN, Gentamicin; IMP, Imipenem; LVX, Levofloxacin; MEM, Meropenem; NIT, Nitrofurantoin; PMB, Polymyxin B; SAM, Ampicillin/sulbactam; SMZ/TMP, Sulfamethoxazole/Trimethoprim; TOB, Tobramycin; TGC, Tigecycline; TZP, Piperacillin/tazobactam. | | | | | | | | | | | | | | | | | | |
