# Supplementary material for: Two New Lytic Bacteriophages of the Myoviridae Family Against Carbapenem-Resistant Acinetobacter baumannii
Source: Front Microbiol. 2018 Apr 30;9:850. doi: 10.3389/fmicb.2018.00850 (PMC5936750; doi:10.3389/fmicb.2018.00850)
Supplement: Supplementary file 2 [file Table_2.DOCX]

**Table S2. Survival rates of *Galleria mellonella***

| **Group** | **Survival% at each interval** | | | | |
| --- | --- | --- | --- | --- | --- |
|  | 0 h | 24 h | 48 h | 72 h | 96 h |
| Ab1186+PBS | 100 | 37.50 | 18.75 | 18.75 | 18.75 |
| Ab1186+WCHABP1 | 100 | 87.50 | 81.25 | 75.00 | 75.00 |
| Ab1186+Polymyxin B | 100 | 50.00 | 43.75 | 37.50 | 25.00 |
| Ab1186+WCHABP1/Polymyxin B | 100 | 81.25 | 68.75 | 68.75 | 68.75 |
| Ab1262+PBS | 100 | 37.50 | 18.75 | 18.75 | 12.50 |
| Ab1262+WCHABP12 | 100 | 87.50 | 81.25 | 75.00 | 75.00 |
| Ab1262+Polymyxin B | 100 | 43.75 | 25.00 | 25.00 | 25.00 |
| Ab1262+WCHABP12/Polymyxin B | 100 | 81.25 | 75.00 | 68.75 | 68.75 |
| WCHABP1 alone | 100 | 100 | 100 | 100 | 100 |
| WCHABP12 alone | 100 | 100 | 100 | 100 | 100 |
| PBS alone | 100 | 100 | 100 | 100 | 100 |
| Polymyxin B alone | 100 | 100 | 100 | 100 | 100 |
