# Supplementary material for: Two New Lytic Bacteriophages of the Myoviridae Family Against Carbapenem-Resistant Acinetobacter baumannii
Source: Front Microbiol. 2018 Apr 30;9:850. doi: 10.3389/fmicb.2018.00850 (PMC5936750; doi:10.3389/fmicb.2018.00850)
Supplement: Supplementary file 3 [file Image_1.PDF]

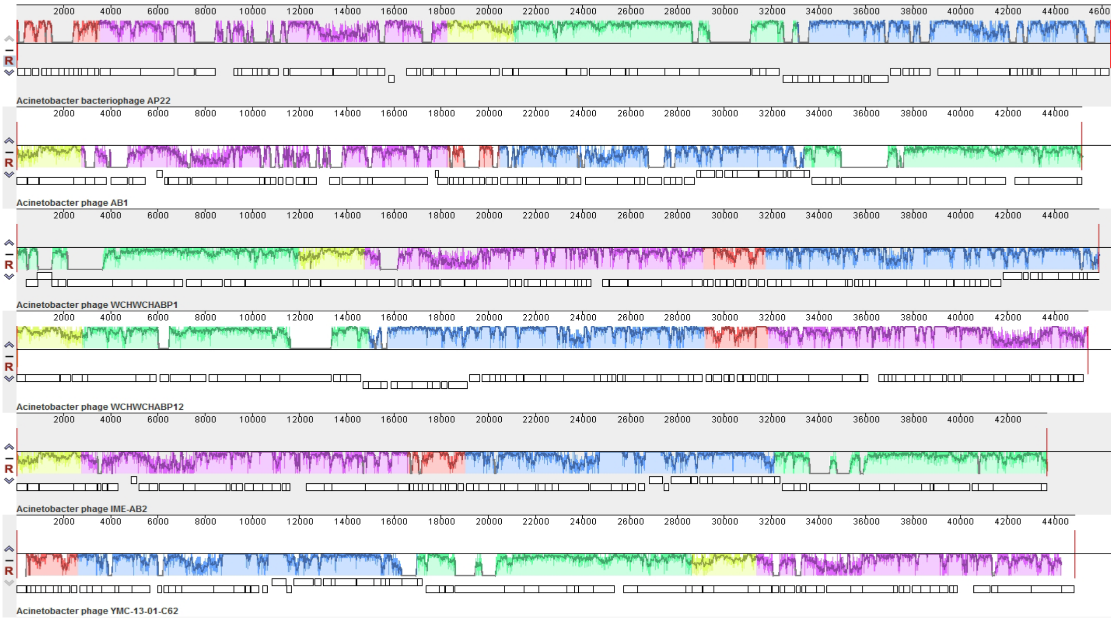

Fig S1. Multiple genome alignments of WCHABP1, WCHABP12 and bacteriophages of the *Ap22virus* genus. Boxes with identical colors represent local colinear blocks (LCB), indicating homologous DNA regions that are shared by two or more genomes without sequence rearrangements. LCBs shown below the horizontal black line represent reverse complements of the reference LCB.

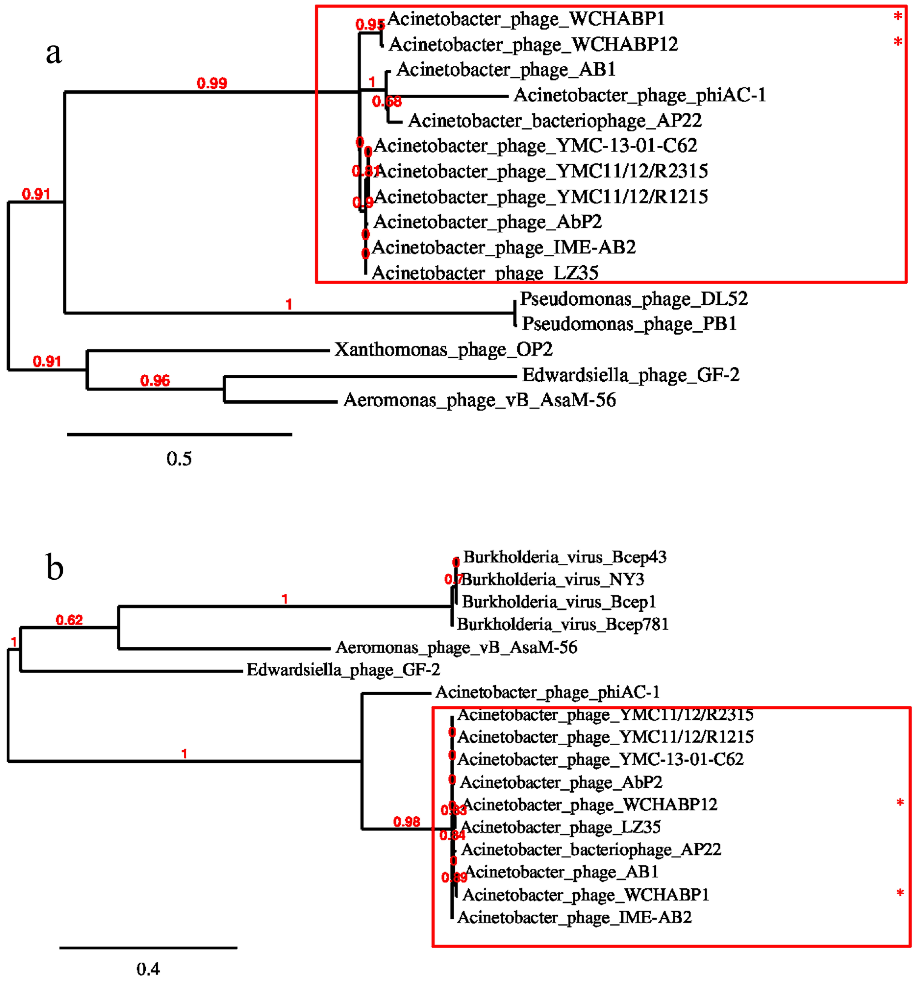

Fig S2. Phylogenetic trees of WCHABP1, WCHABP12 and bacteriophages of the *Ap22virus* genus. a, The tree based on the gene encoding the major capsid protein. b, The tree based on the gene encoding the baseplate J protein. Bar indicates the number of amino acid substitution per site.

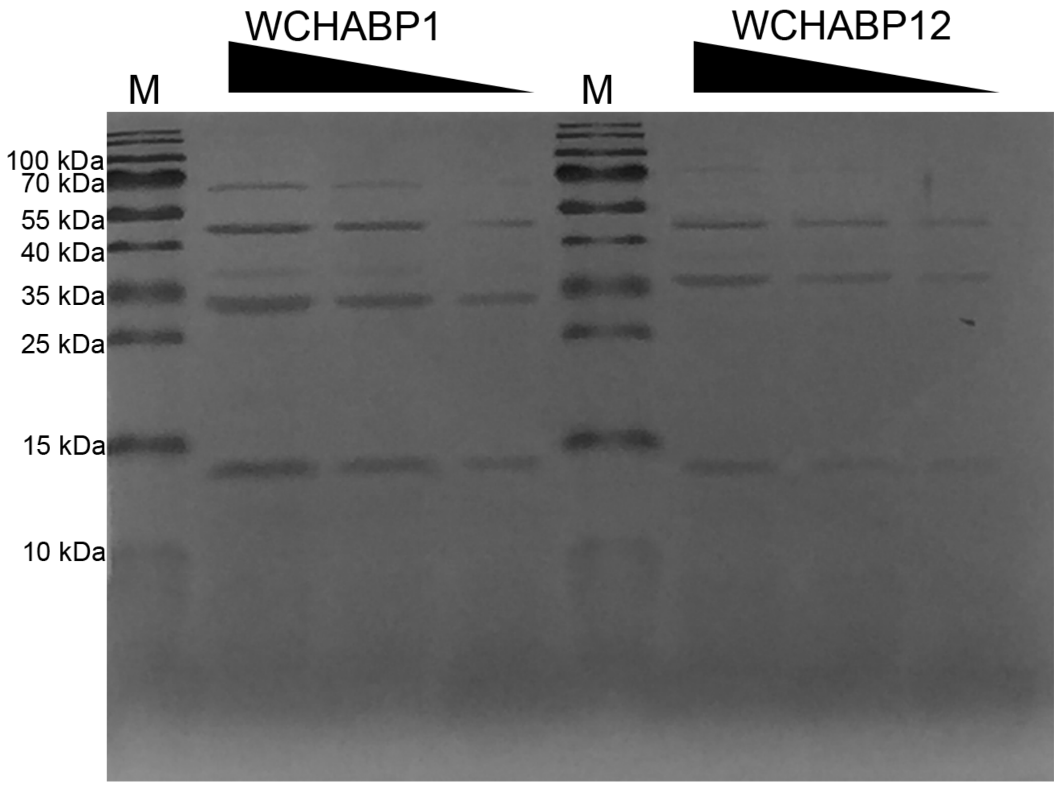

Fig S3. 1-D SDS-PAGE of structural proteins of WCHABP1 and WCHABP2. The triangle represents decreasing concentrations of loaded protein.

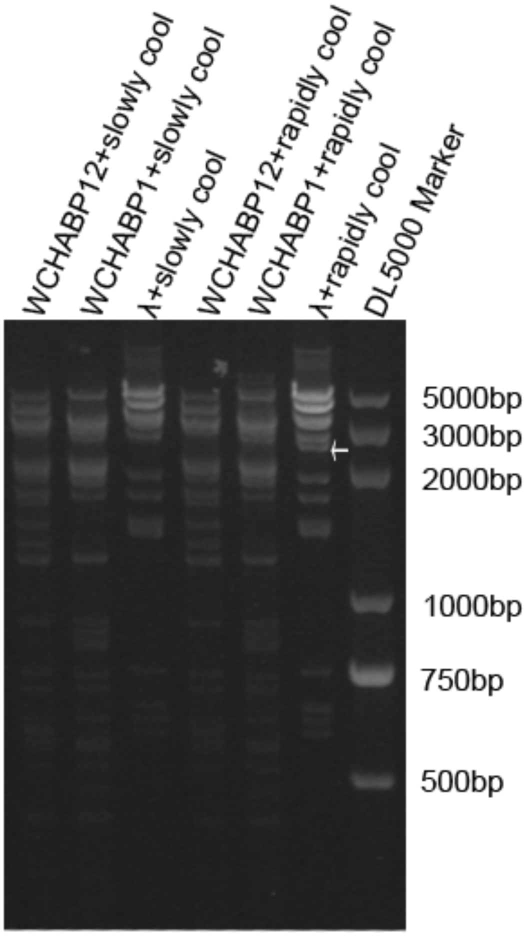

Fig S4. Endonuclease digestion analysis of WCHABP1 and WCHABP12. Genomes of WCHABP1 and WCHABP12 were digested with the restriction enzyme *Hind*III. The digested DNA fragments were separated by 1% agarose gel electrophoresis. *EcoR*V-digested lambda DNA was used as a positive control for cohesive ends. The white arrow indicates the fragment produced by the annealing of cohesive ends under rapid cooling.
